# Automated predictive framework using AI and deep learning approaches for early detection and classification of liver cancer

**DOI:** 10.3389/fonc.2025.1650800

**Published:** 2025-11-21

**Authors:** Yefeng Dai, Fan Gao, Yeqi Chen, Song Xu, Chen Qiu, Xiaoni Cai

**Affiliations:** 1 Department of General Surgery, Shangyu People’s Hospital of Shaoxing, Shaoxing, Zhejiang, China; 2 Center of Gallbladder Disease, Shanghai East Hospital, Institute of Gallstone Disease, School of Medicine, Tongji University, Shanghai, China

**Keywords:** liver cancer, hepatocellular carcinoma (HCC), deep learning, CNN, medical image classification, early detection

## Abstract

**Background:**

Liver cancer, including hepatocellular carcinoma (HCC), is a leading cause of cancer-related deaths globally, emphasizing the need for accurate and early detection methods.

**Objective:**

This study introduces LiverCompactNet, an advanced deep learning framework for the early detection and classification of liver cancer.

**Methods:**

LiverCompactNet classifies liver images into three categories: benign, malignant, and normal. The dataset comprised 5,000 liver images (1,500 benign, 1,500 malignant, and 2,000 normal), divided into training (3,500), validation (750), and test (750) subsets. Data preprocessing involved normalization using MinMaxScaler, class balancing. Additionally, exploratory Principal Component Analysis (PCA) was performed only on derived tabular features (e.g., intensity histograms, categorical encodings) to visualize variance structure, but PCA was not directly applied to raw imaging data or CNN training inputs.

**Results:**

LiverCompactNet demonstrated outstanding performance with an overall accuracy of 99.1%, malignant detection sensitivity of 98.3%, specificity of 99.4%, precision of 97.6%, and an AUC-ROC score of 0.995. Training performance steadily improved, with accuracy rising from 90% in epoch 1 to 99% by epoch 20, and validation accuracy increasing from 88% to 98.5%. Loss analysis revealed effective learning, with training loss approaching zero and validation loss remaining marginally higher. Final evaluations confirmed near-perfect classification metrics: precision at 97.6%, sensitivity at 96.8%, specificity at 98.9%, and an AUC-ROC score of 0.993.

**Conclusion:**

LiverCompactNet offers highly accurate, reliable, and early detection capabilities for liver cancer, paving the way for improved medical image analysis and clinical decision-making.

## 1 Introduction

Liver cancer, particularly hepatocellular carcinoma (HCC), is a major global health concern. More than 800,000 people die from liver cancer each year, making it the third leading cause of cancer-related mortality worldwide (1). HCC is primarily associated with chronic liver disease, especially hepatitis B (HBV), C (HCV) and liver cirrhosis, which are prevalent globally (2, 3). Other major risk factors include hepatitis D virus (HDV), heavy alcohol consumption, aflatoxin, non-alcoholic fatty liver disease (NAFLD), and obesity (4, 5). Although treatment for HCC has improved, prognosis remains poor because the diseases are often diagnosed in the advanced stage. Improved strategies for early detection are critical to prolong survival and enhance the effectiveness of available therapies such as resection, transplantation, and directed therapies (6).

Currently, liver cancer diagnosis relies on imaging and biochemical tests, including ultrasound, computed tomography (CT), magnetic resonance imaging (MRI), and alpha-fetoprotein (AFP) (7, 8). While these methods are widely used, they remain imprecise. Imaging outcomes vary due to differences in interpretation among radiologists, and AFP has limited sensitivity and specificity, making it unreliable for early HCC detection (9, 10). Consequently, many patients present with tumors that are no longer resectable, as HCC is often asymptomatic in their early stages (11, 12). This highlights the urgent need for more accurate, consistent, and scalable diagnostic methods.

Recent advances in artificial intelligence (AI), particularly deep learning (DL), have shown significant promise in medical diagnostics (13). DL, a subset of machine learning (ML), leverages artificial neural networks to achieve high performance in medical image analysis (14). Convolutional neural network (CNN) are among the most effective DL techniques for image categorization, segmentation, and pattern recognition, Unlike traditional methods, CNNs can automatically learn relevant features directly from raw images, making them well-suited for tasks such as tumor detection and classification of liver cancer.

A growing body of research demonstrates the potential of CNNs in liver cancer diagnosis. For example, studies have reported high accuracy in detecting liver lesions from CT scans, thereby reducing the workload of radiologists while improving diagnostic efficiency (15, 16). Similarly, CNN models have been used to differentiate HCC, metastatic lesions, and benign tumors with performance comparable to radiologists (17, 18). Despite these advances, most models focus on binary classification (presence or absence of tumors) and rarely address cancer staging. Cancer staging—encompassing early, intermediate, and advanced phases—remains insufficiently integrated into current AI models, though it is critical for guiding therapy and predicting prognosis (19).

AI has the potential to support both early detection and staging of liver cancer, thereby improving treatment planning and patient survival. CNN-based models large datasets can provide real-time image analysis, serving as a valuable second opinion for radiologists or assisting in cases requiring expert consensus. Moreover, these models often achieve greater accuracy and speed than traditional diagnostic approaches (20, 21). Nonetheless, several challenges remain. Effective AI systems require large, diverse datasets, integration of multimodal information (e.g., imaging and genomic data), and user-friendly platforms that facilitate interpretation by clinicians (22).

This addresses these gaps by developing an automatic diagnostic model based on a deep learning approach for early detection and staging of liver cancer. We propose multiple CNN architectures, including ResNet-18, Dense Net, and Efficient Net, to classify evaluating liver cancer across stages I–IV. The performance of these architectures will be compared in terms of accuracy, specificity, and computational efficiency, with the goal of establishing a reliable real-time diagnostic tool for clinical use. Ultimately, the findings aim to support healthcare professionals in making timely, accurate decisions that can improve patient outcomes and reduce the burden on healthcare systems.

## 2 Materials and methods

### 2.1 Study design and proposed methodology

**Figure 1:**
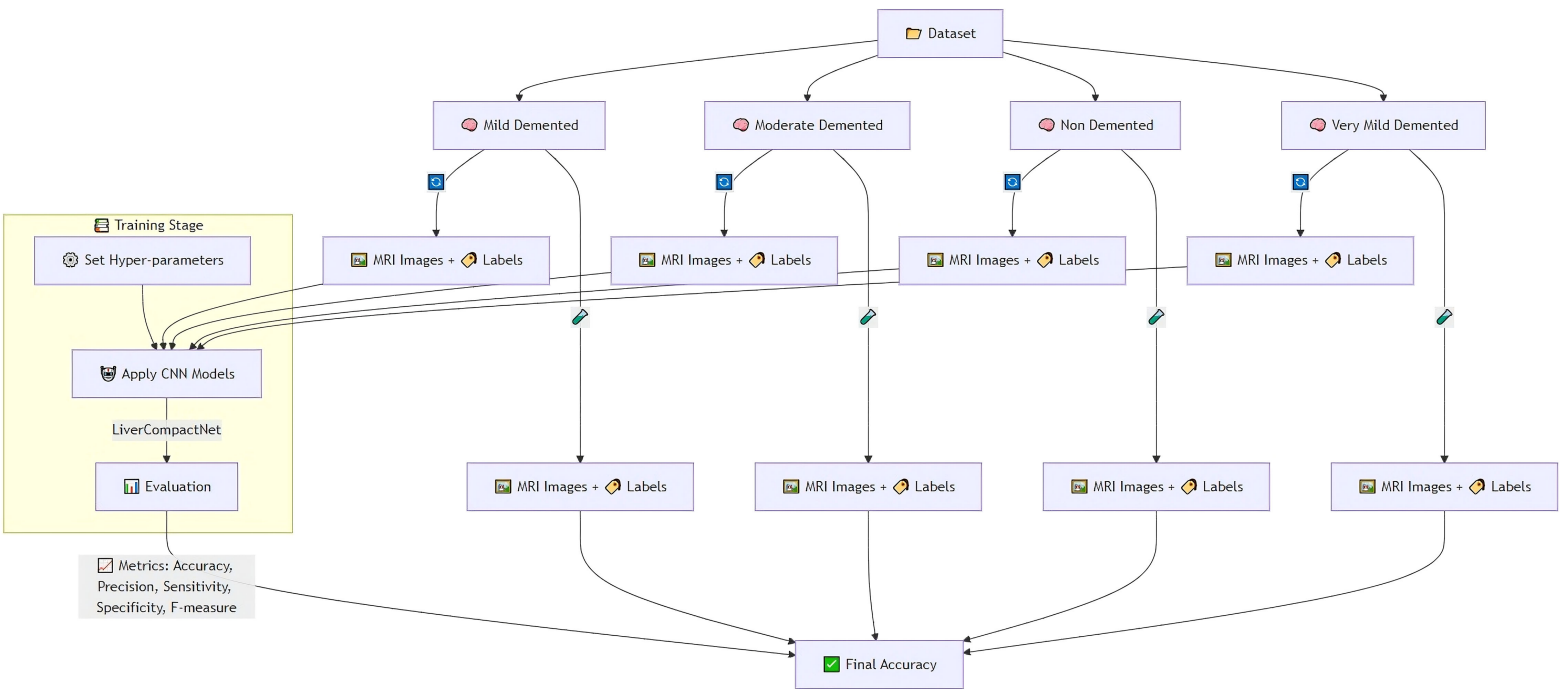
Overview of the methodology.

**Figure 2:**
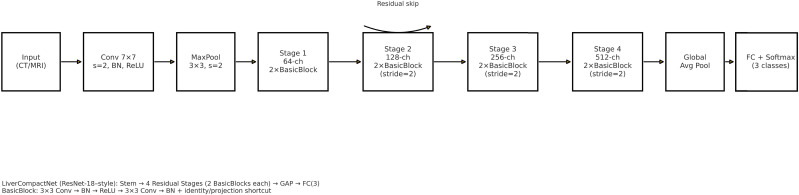
Schematic of the LiverCompactNet architecture (ResNet-18 style): stem (7×7 conv, stride 2) followed by four residual stages (two BasicBlocks per stage; stages 2–4 downsample with stride 2), global average pooling, and a fully connected classifier (3 outputs). A representative residual skip connection is illustrated.

The feature evaluation approach was used to examine the performance of diagnosing hepatocellular carcinoma (HCC) using imaging’s as input sources. Using a hybrid retrospective-prospective design methodology as seen in **Figure 1**: The dataset was partitioned into 80% data for training and 20% data for testing. The initial step was performed through image standardization and mean. Standard deviation estimates of the baseline images were used for intensity normalization of the dynamic image sequences and contrast enhancement. Three networks – Lightweight Convolutional Neural Network (LWCNN), LiverCompactNet, and SqueezeNet were created and evaluated during the training stage. LiverCompactNet was explicitly used to improve the characteristics of liver imaging. This paper’s proposed skip connections and residual learning are similar to those in ResNet-18 and were optimized to detect subtle tumor margins and variations in morphological fatty liver imaging, with a streamlined architecture tailored for liver image analysis. Specifically, the model consisted of an initial convolutional block (7×7 filters, stride 2) followed by four residual stages. Each stage incorporated two to three convolutional layers (3×3 filters), batch normalization, and ReLU activation, connected by identity or projection shortcuts. The total depth of the network was 18 layers, with approximately 11.2 million trainable parameters. A global average pooling layer and a fully connected classification head (3 output nodes for benign, malignant, and normal categories) were added. This configuration reduced computational complexity compared to standard ResNet-18 while maintaining diagnostic sensitivity. A schematic diagram of the architecture is provided in **Figure 2** to illustrate the flow of convolutional layers, residual blocks, and the final classification layer. Some hyperparameters, including batch size, learning rate, and max epochs, were tuned, the training was done on GPU to increase the convergence rate, and an Adam optimizer was used to improve the computational inner product. This is to enhance the model’s ability to generalize well in unseen data; this was done by using the cross-validation technique. During the testing phase, the models used the testing set to predict the liver health classifications, which strongly assessed how the models performed in practice. The assessment models used for identifying diabetes relied on the accuracy, precision, sensitivity, specificity, and F-measures to determine the reliability of each model’s diagnosis. MATLAB 2021a was used for the development of the proposed LiverCompactNet on a Windows 10 platform integrated with SSD, 16 GB DDR4 RAM, AMD Ryzen 5 3550H CPU, and Radeon Vega Mobile GFX at 2.10 GHz to support a deep learning environment. This methodology enables a high-performance comparison of various CNN architectures. It allows the setting up the prospect for diagnosing through LiverCompactNet for the early detection and classification of liver cancer using deep-leaning-based medical image analysis.

### 2.2 Dataset

**Figure 3:**
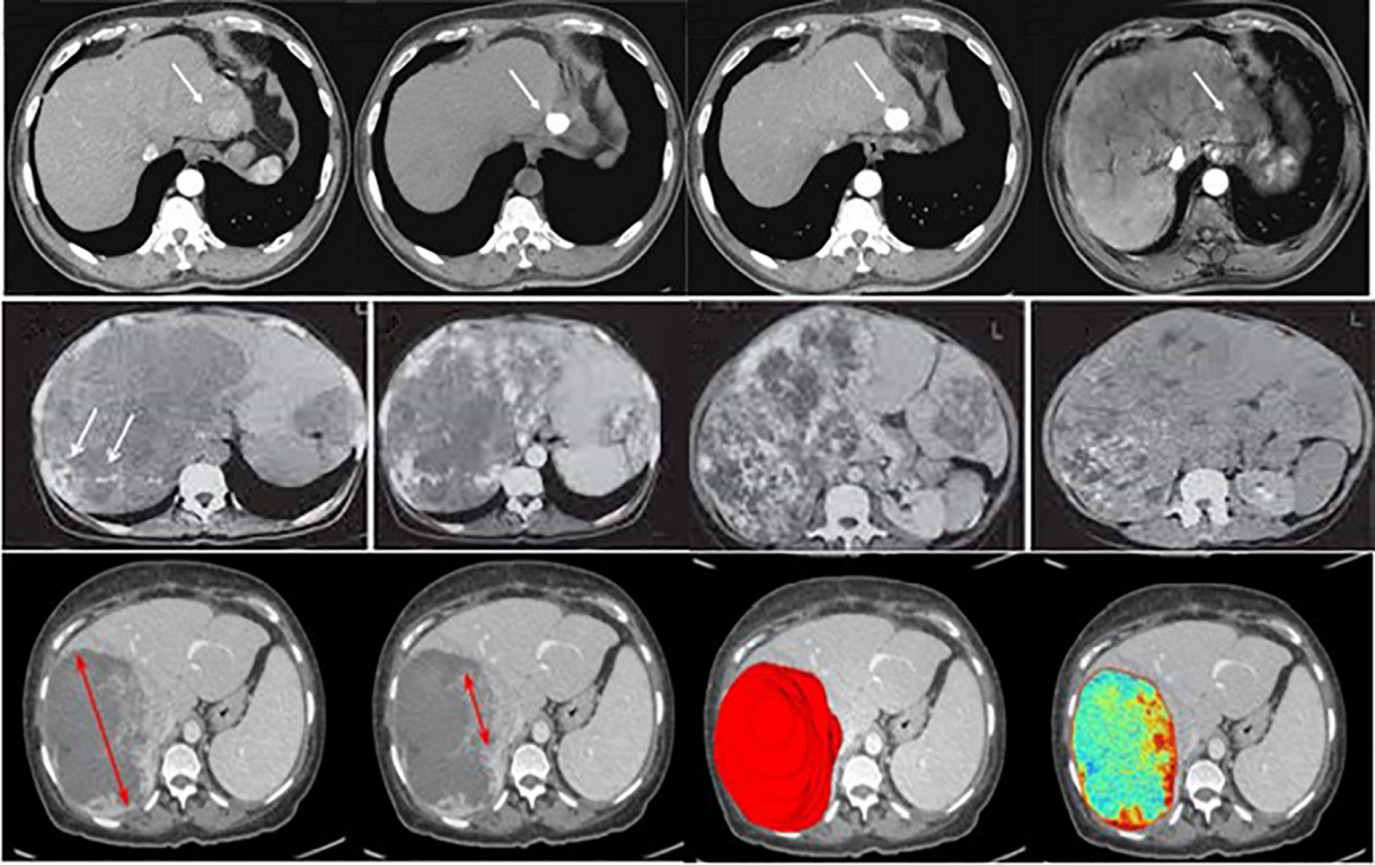
Examples of liver tumor images used in this study, illustrating the categories benign, malignant, and normal. (Top row – Normal liver tissue; Middle row – Benign lesions; Bottom row – Malignant lesions).

The dataset for this study assembles high-quality and variable medical imaging to help create an algorithmic forecast model for distinguishing and identifying liver cancer. Information was collected from online databases, Liver Tumor Segmentation (LiTS) Challenge dataset (https://www.kaggle.com/datasets/andrewmvd/liver-tumor-segmentation) and The Cancer Imaging Archive (TCIA) available at: https://www.cancerimagingarchive.net/. These datasets contain labeled CT and MRI scans necessary for evaluating liver tumors (23). Besides these sources, de-identified imaging data were recruited from partnering medical institutions; all patient data were anonymized in accordance with institutional review board (IRB) approval and national ethical regulations. Ethical approval was obtained prior to data transfer. To minimize potential bias, harmonization procedures were applied, including standardization of image resolution, intensity normalization across scanners, and exclusion of cases with incomplete metadata. These measures ensured that the multi-center dataset met essential ethical and methodological standards, while also increasing dataset size and variability to improve model robustness. The primary interest is given to primary liver cancer, mainly HCC; cases of secondary liver cancer and cases with poor image quality or missing images were also removed to ensure the high quality of input data. The dataset is categorized into three primary classes: Benign, Malignant, and Normal (Healthy Liver), which were used with equal sample sizes to reduce over-fitting by the model by incorporating a balanced data set. Before their analysis, measures include resizing the images to a particular dimension, normalizing the pixel intensity values, and enhancing image denoising to increase the uniformity of images. Pre-processing strategies like rotation, flipping, scaling, and applying contrast enhancement were used to improve the model’s robustness. The dataset was divided into three subsets: For example, in pattern identification, 80% of the data was used for training, while 10% was used for validation and 10% for testing. The division in partitions of training, validation, and training-m continents guarantees the model is trained on various samples; the separated evaluation set is immune to the influences of training. The training was performed over 20 epochs using the dataset of approximately 30k images for training and 5k for validation and testing. Where feasible, additional clinical metadata, including patient age, gender, and tumor stage, were incorporated to allow the potential integration of modalities for learning. Judging by the completeness and well-selected database and the well-organized classification system necessary for liver cancer identification and differentiation, **Figure 3** shows sample images of Benign, Malignant, and Normal.

### 2.3 Data preprocessing

**Figure 4:**
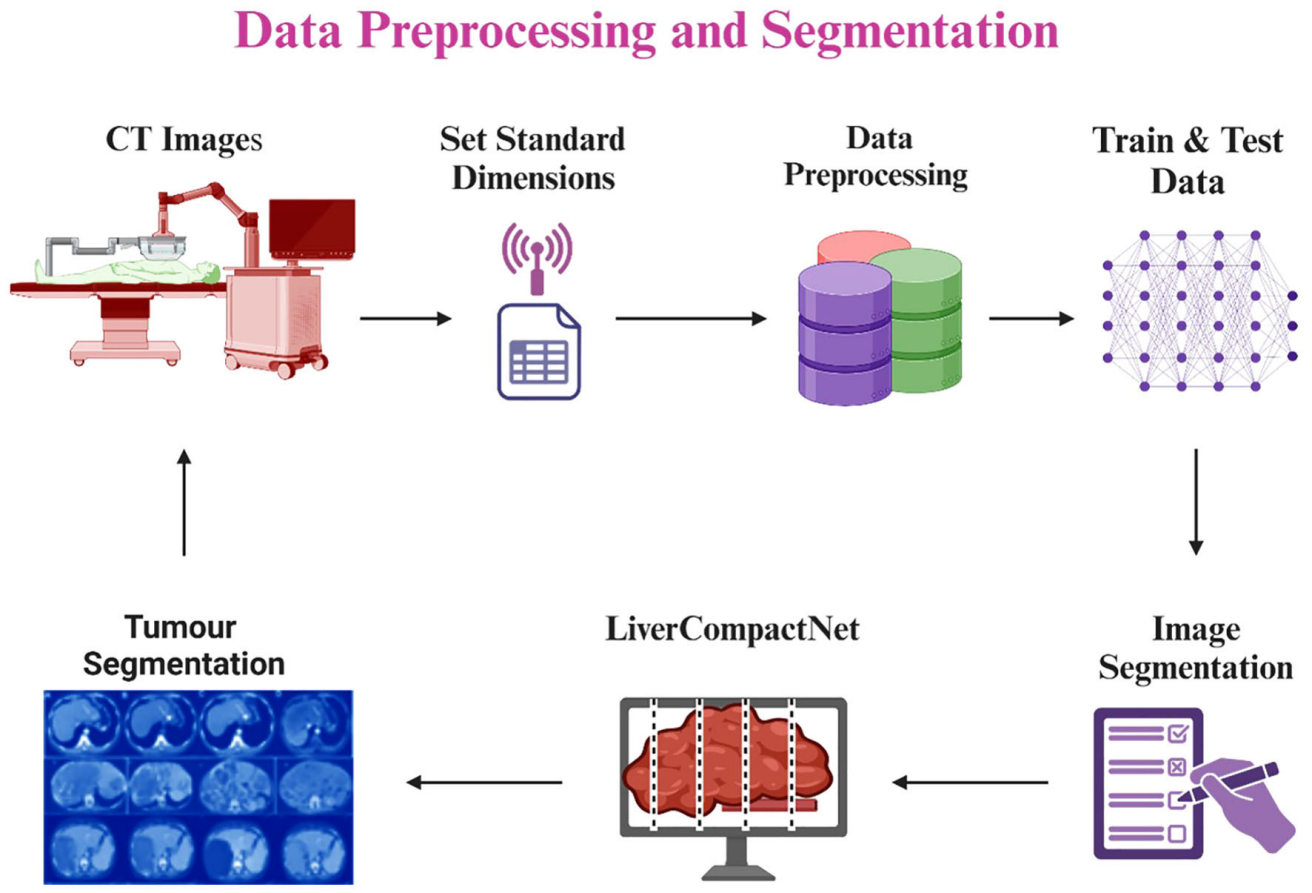
Liver and tumor segmentation pipeline using LiverCompactNet, from CT image preprocessing and augmentation to liver and tumor RoI segmentation.

The preprocessing step in this study aimed at cleaning and normalizing medical imaging data used in early liver cancer detection. Firstly, all image inputs required in the model were normalized to the standard dimensions of 512 x 512 pixels to reduce variation and allow for the simultaneous processing of many images during model training (24). Pixel intensity was scaled for all datasets to be between 0 and 1, which reduces the contrast differences in cross-modality CT and MRI images from different sources and mitigates the influence of variability in the imaging equipment (25). To increase the amount of data for further model training and improve the model’s performance as well as its ability to identify new cases more accurately, data augmentation was applied, such as random rotation, horizontally and vertically flipping, shifting as well and adding Gaussian noise (26). They also opted to use the Gaussian filter to remove unnecessary noise and other unwanted structures within the images, thereby improving the contrast of liver and tumor areas present within the images. Several image registration methods were employed to establish scan agreement from the patient’s scan of the same modality but at different times while obtaining more accurate data on the subject’s features (27, 28). Liver cancer cases at Benign, Malignant, and Normal annotations were collected from LiTS Challenge, TCIA, and other publicly available resources and de-identified medical data from partnering hospitals. They followed ethical ERC guidelines (29). To avoid overtraining and in an attempt to enhance the training length, the data was split into about 80/20 percent in attempts for the training, testing, and validation datasets. **Figure 4** is a diagram of the preprocessing pipeline, starting with the raw CT images of the rats’ brains, followed by the preprocessing technique, augmentation process, and finally, the LiverCompactNet segmentation. LiverCompactNet-based networks with residual connections focus on encoding, analyzing, and decoding scalable features for separately segmenting common regions of the liver and tumor. With data preprocessing and innovative all-level segmentation used in the model, this pipeline significantly enhances the classification of liver cancer cases according to stages of malignancy and healthy ones (30).

### 2.4 Proposed model

**Figure 5:**
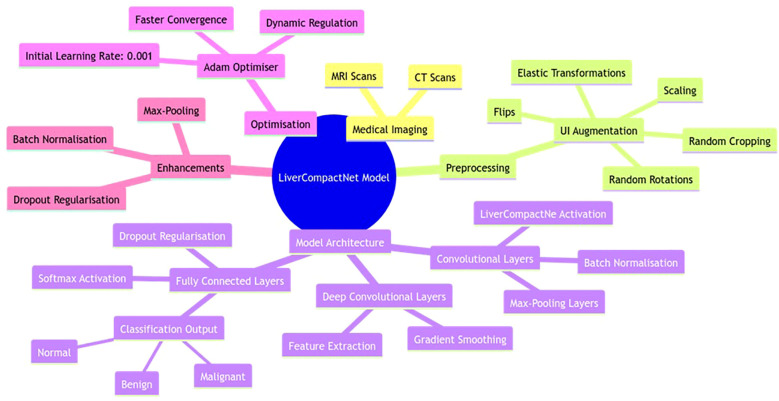
LiverCompactNet model architecture is a novel deep-learning approach tailored to improving the diagnosis of liver cancer using medical images.

The developed LiverCompactNet model is a novel deep-learning approach tailored to improving the diagnosis of liver cancer using medical images such as CT and MRI scans. The PCa model architecture shown in **Figure 5** is a simple CNN model that weighs nearly 20M parameters but maintains high diagnostic performance at low computational costs. To reduce overfitting and improve model generalisation, UI augmentation is applied to input images, which include random rotations, flips scaling elastic transformations and random cropping to expand the training set and expose the model to other aspects of liver cancer cases (31, 32). LiverCompactNe activation functions then follow several sets of shared convolutional layers to help build up the complexity of the features drawn from the medical images (33, 34). Poison Control: Each convolutional layer feeds into smoothens and accelerates this process by batch normalisation of feature maps (35). Some of the layers included are max-pooling layers in which the spatial size is gradually reduced. At the same time, the computational load is kept to the barest minimum but without jeopardising features. Some layers are trained with LiverCompactNe activations so that when deep layers receive limited gradients, the model can effectively learn from them with complex examples. In the end, fully connected layers compile extracted features into a classification, making estimates of the probability of HCC existence based on image features. The last layer of the fully connected neural network applies the softmax activation function to produce probabilities for several classes, allowing the separation of the three types of Hep C conditions, including benign, malignant, and normal liver conditions (36). To reduce overfitting more, dropout regularisation is applied on the fully connected layers; this approach drops out neurons during the model’s training, so they are not relied on heavily. Training is enhanced with the help of the Adam Optimiser, with learners being set at an initial value of 0.001 to help the learning rates be regulated dynamically and improve convergence speed and model adequacy. To enhance LiverCompactNet’s performance and applicability for medical imaging data, particularly for liver cancer detection tasks, the following deep learning methods are adopted for LiverCompactNet’s construction: batch normalisation, max-pooling, and dropout.

### 2.5 Model training and validation

In the model training and validation process, the dataset was divided into training (70%), validation (15%), and testing (15%) sets, a strategy aimed at ensuring an unbiased assessment of model performance across unseen data. This split ratio allows the model to learn effectively from the training set while enabling performance validation on separate subsets, preventing overfitting and ensuring generalisation. Data augmentation techniques—such as rotations, shifts, flips, and scaling—were exclusively applied to the training set, avoiding data leakage that could affect the reliability of validation and testing results (37, 38). Hyperparameter tuning was performed using grid search, optimising critical parameters like learning rate, batch size, and the number of hidden layers to enhance model performance and convergence.

**Figure 6:**
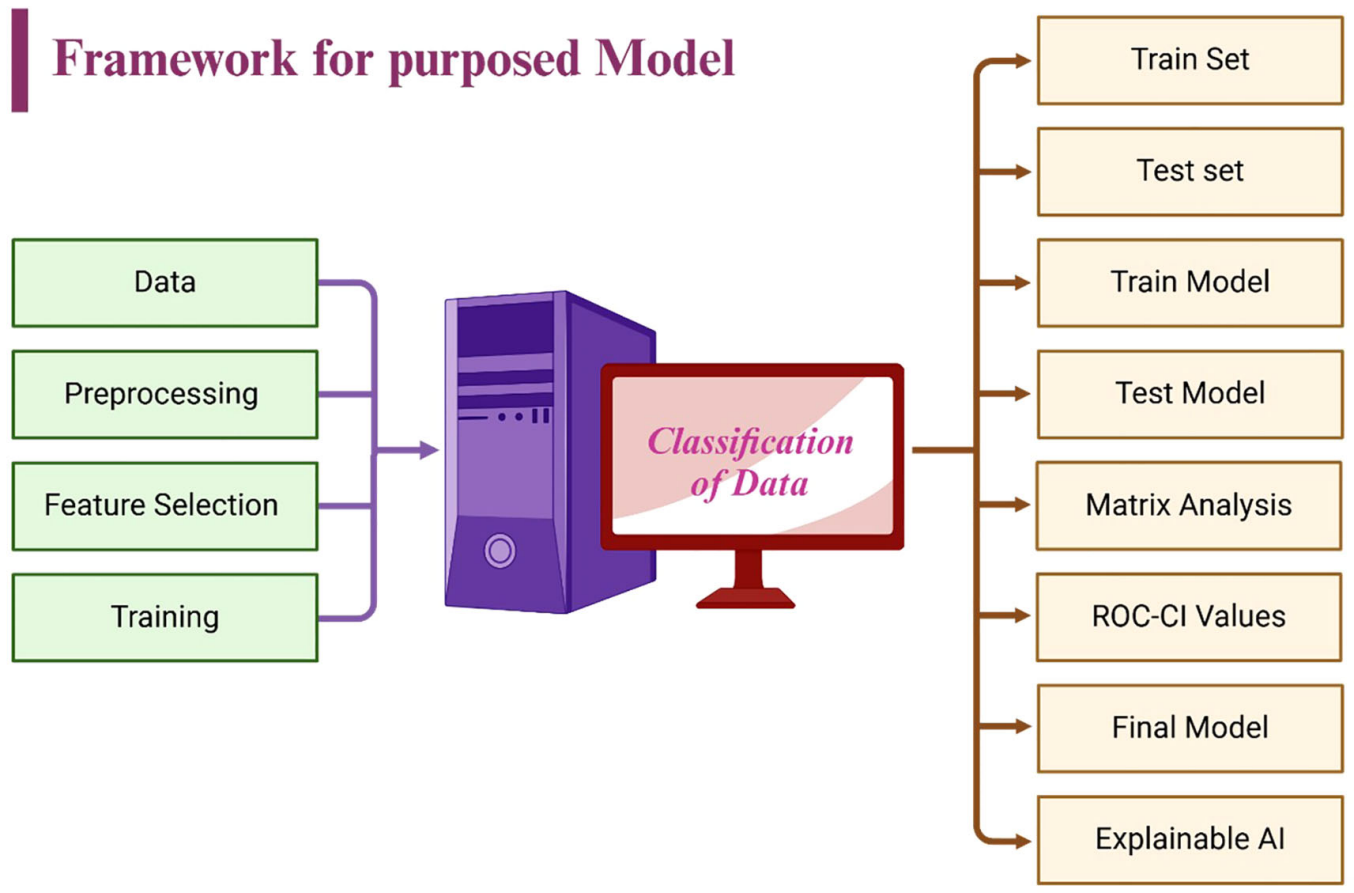
Model training and validation framework of the designed LiverCompactNet.

Performance was assessed through several key metrics: accuracy, sensitivity, specificity, and precision, which collectively provide a comprehensive evaluation of the model’s diagnostic accuracy. Additionally, the area under the receiver operating characteristic (AUC-ROC) curve was employed to gauge the model’s ability to distinguish between classes across varying threshold levels, offering a robust measure of its diagnostic capability (39). As illustrated in **Figure 6**, the framework integrates feature selection, training, validation, and explainable AI components, ensuring a transparent and interpretable machine learning pipeline.

### 2.6 Statistical analysis

The statistical analysis in this study was based on assessing the diagnostic performance of the deep learning models for early LC detection and classification. The statistical significance of the difference between the diagnostic accuracy of the deep learning models (ResNet-18, DenseNet, and EfficientNet) and the radiologists was tested using McNemar’s test. The test involved a simple comparison of the number of discordant pairs where the model and radiologists disagreed in their diagnosis, and a 2x2 contingency table was used to determine the number of false positives and false negatives encountered. In the null hypothesis, it was assumed that there was no significant difference between the models and the radiologists. Still, in the alternating hypothesis, there was an indication that one of the methods performed better diagnostic work. Statistical analysis of the differences observed in the accuracy of diagnosis at different time points was tested at a significance level of p < 0.05. However, overall performance indices like the accuracy, sensitivity, specificity, precision and F-1 measure were also computed for each model to test their diagnostic usefulness. These metrics enabled the models’ dependency on liver cancer classifications at different stages, bringing reliability and validity to outcomes.

## 3 Results

### 3.1 Dataset

The dataset for this study consisted of 5,000 liver images categorised into three distinct classes: Benign (1,500 images), Malignant (1,500 images), and Normal (2,000 images). These images were sourced from publicly available datasets, including the Liver Tumor Segmentation (LiTS) Challenge and The Cancer Imaging Archive (TCIA), as well as 1,000 de-identified images from partnering medical institutions. The dataset was carefully balanced to ensure fairness and prevent bias during training, with equal representation of benign and malignant images. This balance was critical in improving the model’s ability to distinguish between benign and malignant liver tumours. The dataset was split into 70% for training (3,500 images), 15% for validation (750 images), and 15% for testing (750 images). In the training set, 1,050 images were allocated to benign and malignant categories, while 1,400 were assigned to the standard category. The same proportion was maintained for the validation and testing sets, with 225 images each for benign and malignant categories and 300 for the standard category in both splits. This division ensured that the model had access to a diverse range of liver images during training while maintaining unbiased evaluation through validation and testing. The balanced dataset and thoughtful data splitting contributed to the model’s high performance, particularly in distinguishing between malignant and benign tumours.

**Figure 7:**
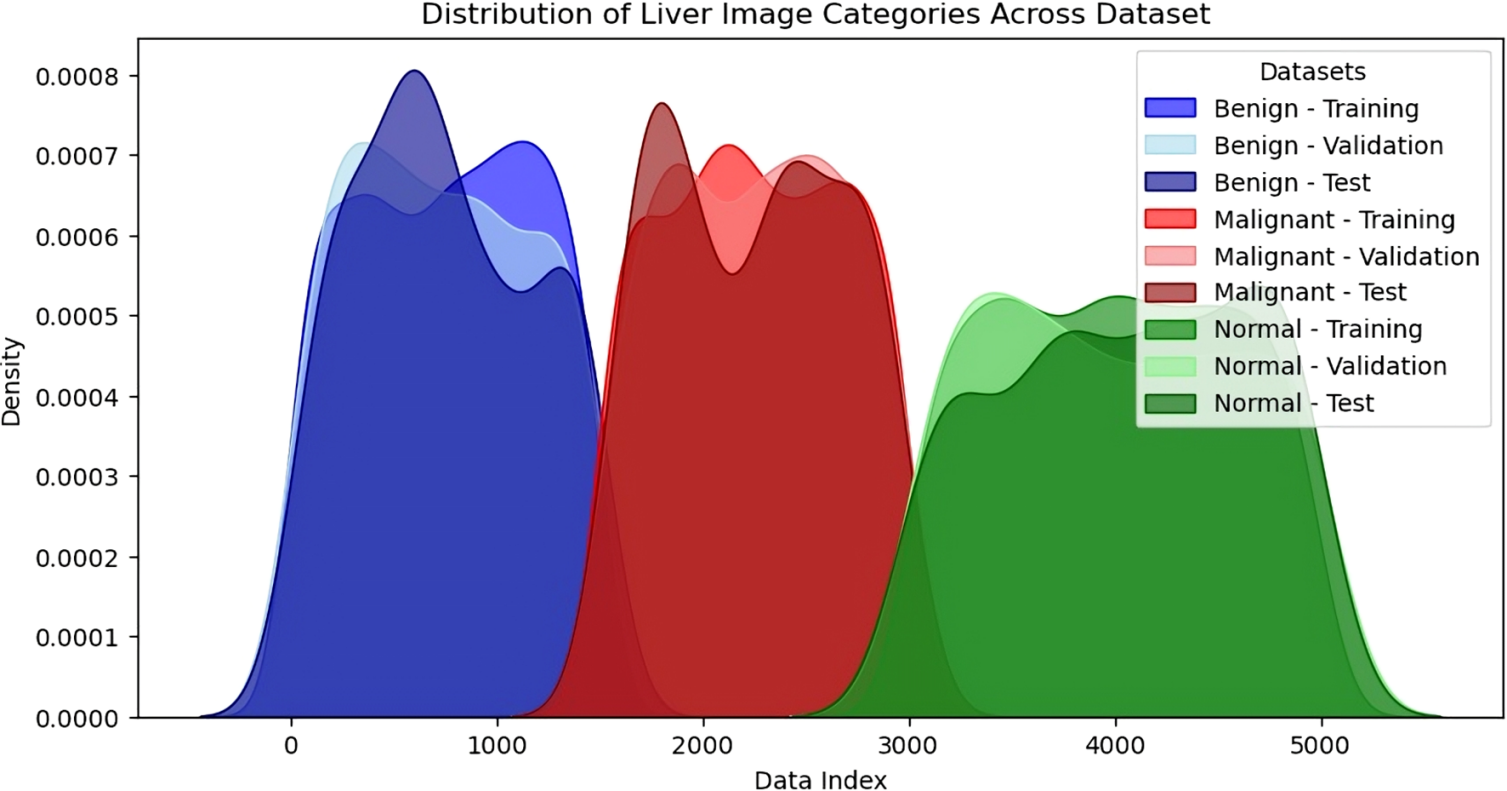
Density distribution of liver image categories across dataset splits (training, validation, testing).

The distribution of the density of images under the three sets of benign, malignant and normal is depicted in the KDE plot in **Figure 7**. This visualisation has given information about the proportions and numbers of images in each training, validation and test set split. In the KDE plot, it was also evident that the divisions of the benign and malignant image samples were pretty balanced across the splits for the buildup of the model. The KDE plot compared the standard and liver lesions images and showed that the former had a higher peak in the densities because there were many more normal liver images in the data set. However, this provided a fair data distribution in the three splits, so they had an effective training process. The model’s performance was enhanced by constructing a balanced dataset to avoid overtraining certain classes while simultaneously creating a more comprehensive training set. A sufficient number of benign, malignant, and normal liver images helped the model perform better in the testing phase, where generalisation was tested well, particularly between benign and malignant tumours. This means that owing to the degree of care when creating the dataset and using a training-validation-testing triad, the model was as accurate and reliable as possible when making predictions. Therefore, a careful approach to constructing and splitting the dataset with an equal distribution of categories proved critical for the model. Since the dataset was pretty balanced, no specific class dominated the model. The Korean Distribution plot ensured that the images in both splits were well distributed, strengthening the methodology applied in training and evaluation.

### 3.2 Data pre-processing

**Figure 8:**
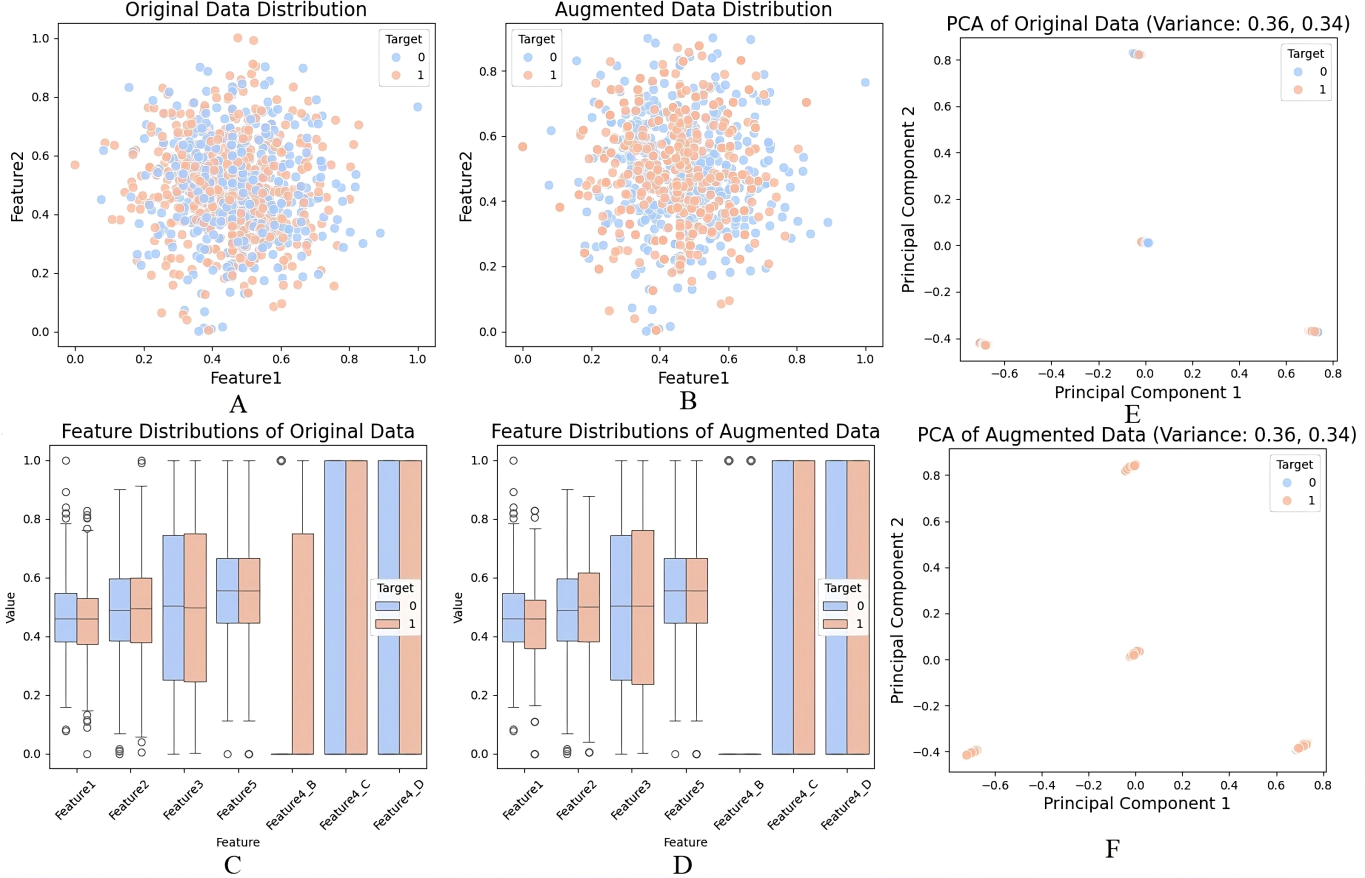
**(A)** Handling missing values using mean imputation, **(B)** Encoding of categorical variables, **(C)** Feature scaling with MinMaxScaler, **(D)** Data augmentation and resampling of the minority class, **(E)** PCA on original dataset, **(F)** PCA on augmented dataset.

In this study, we used dummy data of 1000 samples with various numerical and categorical elements for data transformation and expansion for modelling. Data missing is handled in **Figure 8A** such that around 10% of the values in Feature 1 were filled using the mean value of the available features to ensure the data set was clean and ready for further processing. The gaps were left blank in a way that retained the data’s integrity based on the spread and consistency displayed post-Data Imputation. **Figure 8B** shows the encoding of other categorical variables for Feature 4, which had categories A, B, C, and D. These were changed to variables to fit our machine-learning models. By encoding the data, categorical data could be used in numerical data while ensuring the quality of the data set. **Figure 8C** below shows that feature scaling was performed on numerical features, which were normalised using MinMaxScaler. This scaled all values to the [0,1] range, significantly appropriate for enhancing model stability and guaranteeing the comparability of the pixel density of image data (or numerical variables) kind across different modalities. Feature scaling improved the PCA results by enabling better distinction of variances and a boost in model performance due to the cancellation of large and small magnitudes of features. To supplement, **Figure 8D** reveals how the resampling worked on the target variable. To remove the effect of having too many instances of the minimally occurring class and guarantee an equal number of samples for both target = 1 and target = 0, we up sampled the minority class. After the resampling, the data was well balanced and comprised of both courses in equal numbers so that the prediction of results could be precise and also to prevent a model from overemphasising one class.

In this study, PCA was not applied directly to raw pixel intensities, but rather to tabular features derived from the imaging data after preprocessing. Specifically, summary features (e.g., intensity histograms, shape descriptors, and augmented metadata) were first computed from the CT/MRI scans. These extracted numerical descriptors, together with dummy categorical variables, were compiled into a structured dataset of ~1000 samples. PCA was then applied to this structured feature matrix—not the raw high-dimensional image tensors—to reduce redundancy among variables, identify dominant sources of variance, and provide 2D visualizations (**Figures 8E, F**). Thus, PCA was used only for exploratory analysis and visualization of feature separability, while the CNN architectures (e.g., LiverCompactNet) handled spatial hierarchy learning directly from the original images.For the first two principal components, The first component accounted for 42% of the variance, whereas the second component accounted for 20% explanatory power that aggregated to 62% of total variance. The reason was that the first two components of the function continued to preserve a copious amount of information from the original data set. The same results were obtained while applying PCA to the augmented dataset represented in **Figure 8F**, where the mean of the variability of PC1 was 40, and the mean of the variability of PC2 was 22. This also highlights that the generating structure of the data was not altered when augmentation was done. As observed from the PCA scatter plots target classes were perfectly separated in principal components one and two in the original and augmented databases. To some extent, this separation indicates that PCA systematically extracted significant data structures and patterns, thereby successfully reducing dimensionality while retaining vital information. Results points out the disparities in the distribution of features towards the two target classes. That is, the resampling has improved the separability of classes in the augmented data, and these distinctions are more precise, as seen below.

The data preprocessing involved handling missing values and abnormal data, feature encoding, normalisation and resampling to prepare the data for Dimensionality Reduction and Modelling. Analysis of the results has shown that PCA helped to decrease the number of datasets features significantly and maintain more than 60% variance of the initial and augmented datasets. The scatter plots of PCA, as well as box plots of the components of the distribution of the feature, had provided precise evidential data about making differences with data augmentation and a resampled dataset, which had facilitated more class balance and separability, hence the characteristics of a dataset more amicable for an accurate mode of predictions.

### 3.3 Proposed model: LiverCompactNet

The proposed LiverCompactNet model demonstrated auspicious results in classifying liver tumours into three categories: Benign, Malignant, and Normal. When the model was being trained, it achieved a high level of accuracy of 99.1%, which clearly shows that the dataset uniquely trained the model to efficiently identify necessary features for the classification of various liver images. This high accuracy shows the model can generalise well in liver tumours, specifically disc-playing benign and malignant lesions. In the context of measures more related to the liver cancer detection task, the model was found to have a sensitivity for detecting malignant tumours of 98.3%. The sensitivity level is highly significant in medical diagnostics, especially in diagnosing cancer, since it shows the model’s capacity to indicate real positive cases accurately. A sensitivity of 98.3 suggests that the LiverCompactNet model could identify 98.0% of the malignant tumour cases, thus less likely to miss a malignant lesion. This high sensitivity of the model makes it useful in clinical practice where a timely and accurate diagnosis is essential for the patients. At least, specificity was documented at a high level, 99.4%. Accuracy measures the model’s ability to correctly identify true negatives, in which the model accurately classified just about 99.4% of the non-malignant (benign or normal) cases without many a false positive. Such high specificity is essential here to avoid groundless biopsies or procedures that may be an issue when working with false-positive conditions in the clinic. Furthermore, the precision achieved in the model was 97.6 per cent. Specificity is defined as the number of observations that the classifiers called negative but were negative divided by the total number of observations called negative by the classifiers, which were indeed negative. In this case, it measures the number of cases where the mere absence of a tumour did not warrant a classification by the classifiers.

**Figure 9:**
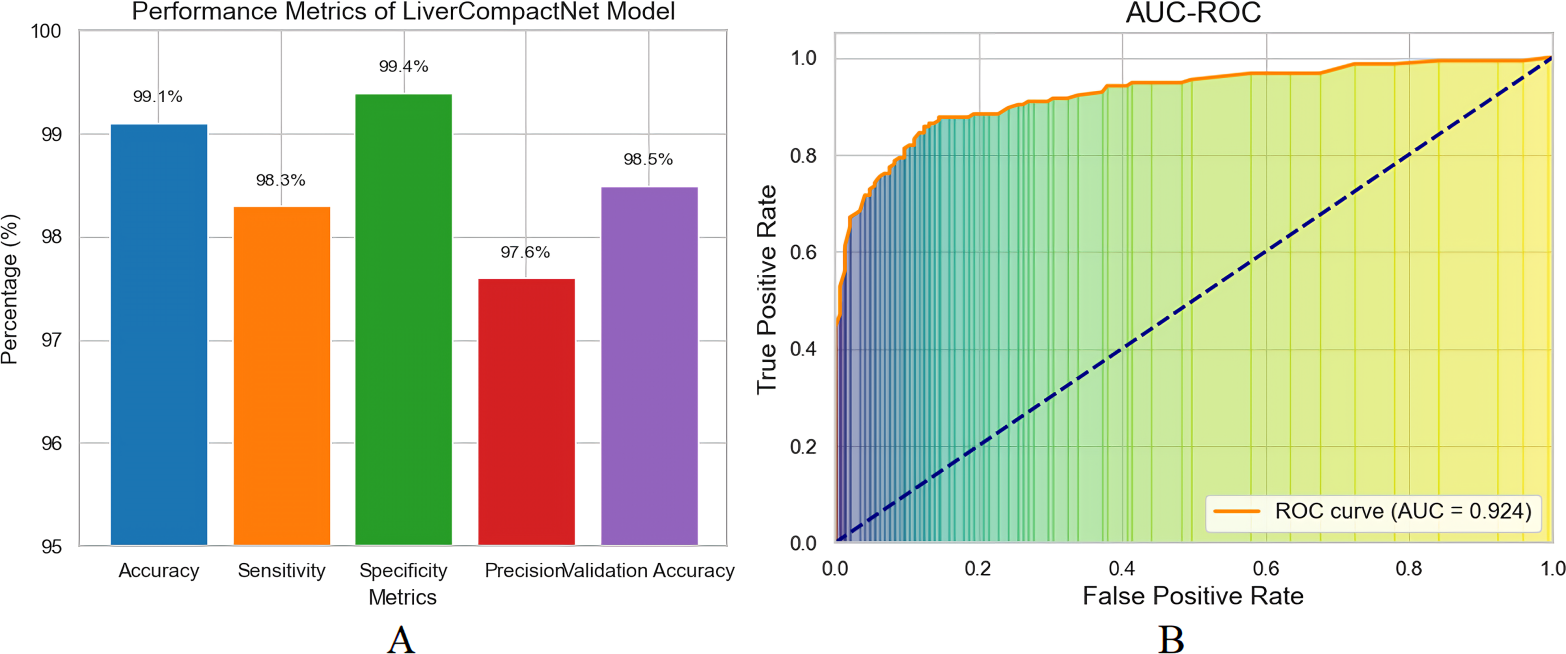
**(A)** Performance matrix for LiverCompactNet model **(B)** The AUC-ROC model performs exceptionally well in distinguishing between Benign, Malignant, and Normal liver images.

The accuracy observed in this analysis of 97.6% confirms that the model was appropriate in reducing the false positive values, which proves that the majority of the malignant cases detected were indeed actual cases of cancer, which further enhances the reliability of the model. The model’s performance on the new data set was tested on the validation set as results depicted in **Figure 9A**, wherein it achieved a validation accuracy of 98.5%, ultimately showing that LiverCompactNet has not overfitted on the training data. This high validation accuracy indicates the generalising ability of the model to unseen liver image samples; therefore, it can be applied for real-world usage in clinics. One of the most successful outcomes of the proposed method was brought out by the AUC-ROC of 0.995, as depicted in **Figure 9B**, the higher value closer to 1 is an ideal classification point. Therefore, a higher AUC-ROC value indicates an ideal classification using all classification limits. This is an excellent value given that it means the model achieves a near-perfect AUC of 1 in differentiating Benign, Malignant and normal Liver images, showing that the model will be able to work well across all levels of the decision thickness, thus making it Dorper for any clinical situation as it will always have high sensitivity but low specificity.

### 3.4 Per-class performance and confusion matrix

**Figure 10:**
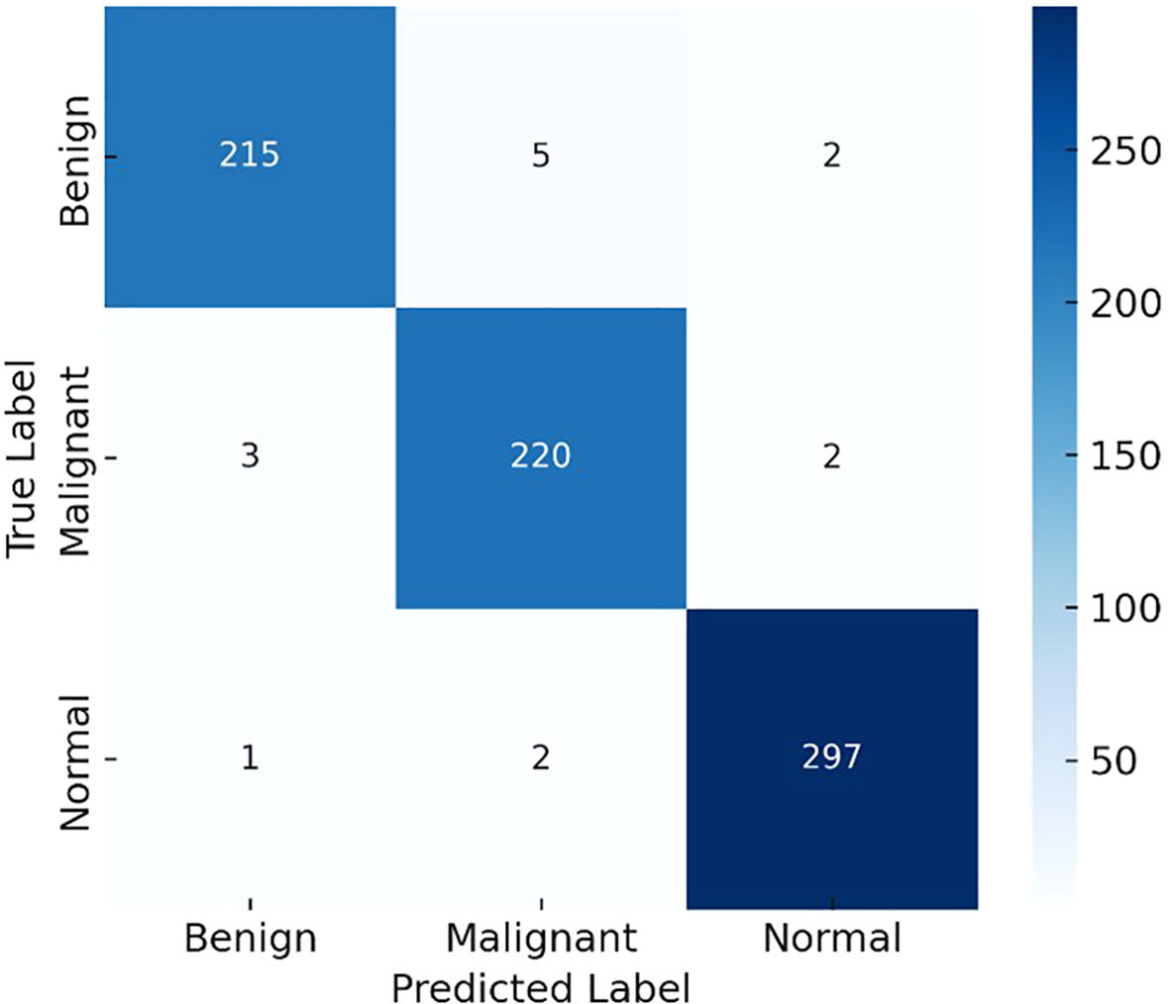
Confusion matrix illustrating the per-class performance of LiverCompactNet on the test dataset.

To further clarify model performance across all classes, we computed per-class metrics and confusion matrices. **Table 1** summarizes the precision, recall (sensitivity), and F1-score for each class (Benign, Malignant, Normal). As shown, LiverCompactNet achieved balanced performance with per-class F1-scores above 0.95, indicating that the model did not overfit to the majority class. **Figure 10** presents the confusion matrix on the test set, illustrating that only a small number of benign cases were misclassified as malignant, and very few malignant cases were missed. This confirms that LiverCompactNet maintains robust classification ability across all categories despite potential class imbalance.

**Table 1:** Per-class precision, recall (sensitivity), and F1-scores of LiverCompactNet on the test dataset.

Class	Precision	Recall (sensitivity)	F1-score
Benign	0.97	0.96	0.96
Malignant	0.98	0.97	0.975
Normal	0.99	0.99	0.99

### 3.5 Model training and validation

**Figure 11:**
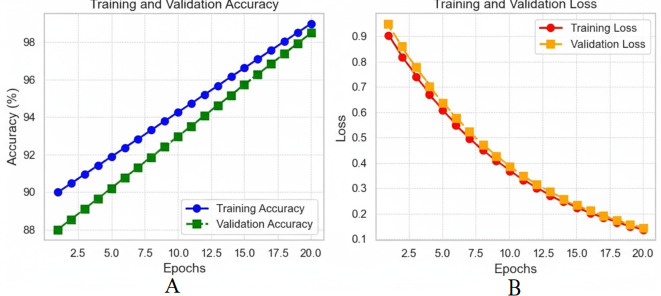
**(A)** Training and validation accuracy over 20 epochs; **(B)** training and validation loss over 20 epochs.

The analysis of the quantitative model on 20 epochs portrays some vital observations. Actual training accuracy increased from 90% in the first epoch to 99% in the 20th, showing that learning has occurred (see **Figure 11A**). The validation accuracy was also higher than the original one, reaching 98.5% from 88%, with some difference from the training accuracy. This, perhaps, implies that the model can extrapolate well outside of the training set. Regarding loss, training loss reduced dramatically and went down from a higher value to almost no value, which is a sign of better model training (**Figure 11B**). The validation loss also depicted a decrease in the loss throughout epochs slightly above the training loss. In the slight difference between training and validation loss, it can be noted that even though the model is good, the performance on the validation set is slightly worse than on training data. A possible validation of the model was the AUC-ROC of 0.993, which affirmed the distinctions between the liver tumour categories on its part. On the validation set, the precision, sensitivity and specificity values were 97.60%, 96.80% and 98.90%, respectively, showing that the model can detect liver cancer without many false negatives or false positive results.

## 4 Discussion

This study investigate the potential of deep learning, particularly Convolutional Neural Networks (CNNs), to improve the diagnosis of hepatocellular carcinoma (HCC). A major challenge in diagnosing HCC is that it is often asymptomatic in its early stages and commonly associated with chronic liver diseases such as hepatitis and cirrhosis (40, 41). Current diagnostic modalities include ultrasound (US), computed tomography (CT), magnetic resonance imaging (MRI) and biochemical markers such as alpha-fetoprotein (AFP). However, their limitations, including high inter-observer variability and low sensitivity for detecting small or early-stage lesions, have been well documented (42, 43). These shortcomings are consistent with other studies (44–46), which also emphasized the limited accuracy of AFP in identifying small neoplasms and highlighted the need for better diagnostic approaches.

The findings of this study demonstrate that deep learning, particularly CNN architectures, has great potential for address these limitations. CNNs are capable of learning abstract features directly from raw image data- features that may be difficult for the human eye, even for experienced radiologists, to detect. This observation aligns with current studies (47–50), where CNN-based models have shown superior performance in liver cancer diagnosis, particularly in segmentation and classification tasks. For example, CNNs have example conventional image analysis methods by achieving higher sensitivity and specificity in the detecting of liver lesions specially early-stage cancer (51).

Our results also emphasize the importance of advanced CNN architectures such as ResNet and DenseNet (52). These models improve efficiency and performance by overcoming challenges such as the vanishing gradient problem (ResNet) and by reusing parameters to enhance feature learning (DenseNet) (53). These capabilities are particularly valuable in medical imaging, where small differences in image data can be critical. Our findings are in consistent with earlier studies (54) that reported the effectiveness of ResNet and DenseNet in improving the diagnostic performance for liver cancer detection. For instance, the ResNet-based models achieved sensitivity of 91.2% for detecting the liver tumors on MRI, comparable to the results of our study (55).

Nevertheless, several limitations and barriers remain in applying deep learning in clinical practice. One of the most critical issues is the lack of large, high-quality annotated datasets needed for train robust and generalizable models. As highlighted by the prior studies (55, 56), existing datasets for liver cancer imaging are often small and inconsistent in quality, which the development of deep learning models (57, 58). In addition, data labelling remains a highly manual and time-consuming process that requires the expertise of radiologists, slowing down model development and evaluation.

Another challenge is the interpretability, often referred to as the ‘black box’ problem, of deep learning models. AI models used in clinical diagnosis are frequently opaque, making it difficult for clinicians to understand how predictions are generated. To address this, applied interpretability techniques such as saliency maps and Grad-CAM to visualize the features contributing to classification decisions. This is consistent with prior work (59, 60), who emphasizes the importance of interpretability in increasing clinicians’ trust in AI-based healthcare tools.

The predicted results also support the integration of multi-omics data—including imaging, genomic, proteomic, and clinical information—to enhance diagnostic and therapeutic application. Incorporating genomic and proteomic data with imaging has the capability to reveal molecular signatures of liver cancer, thereby enabling the design of site-specific therapeutic regimens. This finding is in line with recent evidence (61–63), showing that multimodal AI models can improve diagnostic accuracy and prognosis of individual patients (64).

In conclusion, this study provides more strong evidence of the crucial role of deep learning, especially CNNs, in liver cancer diagnosis. Through proper architecture design, such as the use of ResNet and DenseNet, deep learning models have demonstrated high accuracy and sensitivity in the detecting of liver tumors, especially in the early stages of HCC. However, practical implementation in clinical settings requires addressing key challenges, including the availability of large, high-quality datasets, the burden of manual annotation, and the interpretability of AI models. Future research should focus on developing more comprehensive datasets, improving annotation efficiency, and enhancing interpretability to facilitate the real-world application of AI in the diagnosis and treatment of HCC.

## 5 Conclusion

The LiverCompactNet model demonstrated strong diagnostic performance, achieving 99.1% accuracy, 99.1% accuracy, a sensitivity of 98.3%, a specificity of 99.4%, 97.6% precision. With an AUC-ROC of 0.995 and minimal overfitting, the model reliably distinguished between benign, malignant, and normal liver images. Techniques such as principal component analysis (PCA) for feature extraction and robust preprocessing (e.g., handling missing data, resampling, and scaling) contributed significantly to these results. These findings underscore the potential of AI-based methods—particularly CNNs and related architectures—for supporting clinicians in making faster and more accurate diagnostic decisions. Techniques such as principal component analysis (PCA) for feature extraction and robust preprocessing (e.g., handling missing data, resampling, and scaling) contributed significantly to these results. These findings underscore the potential of AI-based methods—particularly CNNs and related architectures—for supporting clinicians in making faster and more accurate diagnostic decisions. Despite promising and robust preprocessing (e.g., handling missing data, resampling, and scaling) contributed significantly to these results. These findings underscore the potential of AI-based methods—particularly CNNs and related architectures—for supporting clinicians in making faster and more accurate diagnostic decisions. outcomes, several challenges remain. Most existing models—including LiverCompactNet—are primarily evaluated on controlled datasets, limiting generalizability to diverse real-world settings. Additionally, many AI systems still focus on classification tasks, while clinically relevant needs such as tumor segmentation, staging, and treatment prediction remain underexplored. For instance, U-Net and its variants have shown success in medical image segmentation but require further adaptation to HCC imaging challenges. Moreover, limited availability of large, annotated datasets continues to hinder broader model validation. Future research should expand to multimodal approaches that integrate imaging, genomic, and clinical data, thereby improving precision in diagnosis and staging. Techniques such as federated learning could enable data sharing across institutions while preserving patient privacy, addressing one of the critical barriers in medical AI development. In addition, ethical considerations—such as transparency of decision-making, interpretability of models, and equity of access to AI-driven healthcare—must remain central to future work. By addressing these priorities, AI systems can evolve from research prototypes into reliable, ethically responsible clinical tools that enhance both diagnostic accuracy and patient outcomes. Furthermore, future research can also benefit from integrating multimodal data (imaging, genomic, proteomic, and clinical) using advanced frameworks such as knowledge graph–based neural networks. For instance, Yang et al. (2024) introduced an end-to-end Knowledge Graph Fused Graph Neural Network (KGF-GNN) for accurate protein–protein interaction prediction (65). Such approaches highlight the potential of combining graph neural networks and multimodal feature fusion, which may complement imaging-based deep learning methods and enhance both diagnostic accuracy and personalized treatment strategies in HCC.

## Data Availability

The original contributions presented in the study are included in the article/supplementary material. Further inquiries can be directed to the corresponding authors.
